# Heat stress-induced response of the proteomes of leaves from *Salvia splendens* Vista and King

**DOI:** 10.1186/1477-5956-11-25

**Published:** 2013-06-18

**Authors:** Hui Liu, Guozheng Shen, Xianping Fang, Qiaojuan Fu, Kangkang Huang, Yi Chen, Hong Yu, Yun Zhao, Le Zhang, Liang Jin, Songlin Ruan

**Affiliations:** 1 Institute of Horticulture, Hangzhou Academy of Agricultural Sciences, Hangzhou 310024, China; 2 Institute of Biology, Hangzhou Academy of Agricultural Sciences, Hangzhou 310024, China; 3 Institute of Crop Science, College of Agriculture & Biotechnology, Zhejiang University, Hangzhou 310029, China; 4 Experiment Center, Hangzhou Academy of Agricultural Sciences, Hangzhou 310024, China

## Abstract

**Background:**

*Salvia splendens* Ker-Gawl, most commonly used in China to add a splash of brilliant color to the surroundings during the warm season, is subject to heat stress, which can greatly affect its growth and yield.

**Results:**

To gain a comprehensive understanding of heat-tolerance mechanisms of *S. splendens*, we assessed the heat-stress responses and characterized the proteomes of leaves from two varieties, Vista (heat resistant) and King (heat sensitive). Denaturing two-dimensional gel electrophoresis (2–DE) and tandem mass spectrometry were used to identify heat-responsive proteins. Heat stress induced the reversible inactivation of photosystem II reaction centers and increased the amounts of antioxidative enzymes, thereby decreasing oxidative damage. Vista leaves had a much greater ability than King leaves to develop light-protective and oxygen-scavenging systems in response to heat stress. More than 1213 leaf proteome spots were reproducibly detected in the gels, with a total of 33 proteins in each leaf type differentially regulated when *Salvia splendens* were heat stress treated. Of these proteins, 23 and 28 from Vista and King, respectively, were identified.

**Conclusions:**

Most of the identified proteins are involved in photosynthesis, metabolism, protein processing, or stress response, indicating that many different processes work together to establish a new cellular homeostasis in response to heat stress.

## Background

Heat stress is one of the greatest impediments affecting successful development of bedding plants. In general, transitory or consistently high temperatures cause many morpho-anatomical, physiological, and biochemical changes in plants that affect growth and development and thereby drastically reduce yield. Because atmospheric CO_2_ levels have increased since the start of the Industrial Revolution, the average global temperature is expected to increase by 2–5°C during this century [1], which could intensify the effects of heat stress on many plants. Heat tolerance in plants is a complex trait related to morphological and physiological adaptations arising from different genetic characteristics [2]. The resulting damage to plants caused by heat stress includes inhibition of photosynthesis, damage to cell membranes, senescence, and cell death [3]. One mechanism of injury caused by high temperatures involves the overproduction of reactive oxygen species (ROS), e.g., the superoxide radical (O^2–^), the hydroxyl radical (·OH), hydrogen peroxide (H_2_O_2_), and singlet oxygen (^1^O_2_) [4]. ROS can peroxidize membrane lipids, denature proteins, and damage nucleic acids, thereby upsetting cellular homeostasis [5]. However, plants have evolved protective mechanisms to alleviate and repair ROS-induced damage. The major ROS-scavenging mechanisms in nature are enzymatic (superoxide dismutase, SOD; catalase, CAT; peroxidase, POD; ascorbate peroxidase; and glutathione reductase) and non-enzymatic (ascorbic acid and glutathione). Increased expression in the levels of antioxidant enzymes and antioxidants contribute to a plant’s resistance to heat, as reviewed by Almeselmani and colleagues [6].

In addition to the aforementioned protective mechanisms, heat tolerance by plants may partially be the result of an evolutionarily thermally stabilized photosynthetic apparatus including photosystem II (PSII). Stabilization of photosynthetic components is dependent on thylakoid-membrane stability, which varies widely among species and is modulated by acclimation of PSII to heat stress [7]. A direct correlation between membrane stability and yield has been reported for two heat-stressed spring wheat (*Triticum aestivum* L) populations [8]. Another interesting, but still poorly understood aspect of the heat-stress response, is expression of heat-shock proteins (Hsps). These proteins, functioning as chaperones and/or proteases, which are responsible for protein folding, assembly, translocation and degradation in many normal cellular processes, stabilize proteins and membranes, and can assist in protein refolding under stress conditions [9]. As a result, they apparently help prevent and/or minimize the deleterious effects of heat stress at the cellular and molecular levels in plants [10] and other organisms [11,12].

Among a wide variety of bedding plants, *Salvia splendens* Ker-Gawl, belonging to family *Lamiaceae*, is now most commonly used in China to add brilliant color to the environment during the warm season [13]. *S. splendens* Ker-Gawl is therefore grown when it can encounter heat stress. Its growth is greatly affected by temperatures >35°C. Furthermore, only a few heat-tolerant *S. splendens* varieties exist [14]. In short, the limited availability of heat-tolerant *S. splendens* cultivars greatly affects its use as a decorative plant. To date, there have been only a few reports on how *S. splendens* varieties respond to heat stress [13] and no studies that delineate the changes in the proteomic profiles of heat-stressed *S. splendens*. To know better the relatively poor responses of *S. splendens* to heat, which in turn would facilitate the production of heat-resistant *S. splendens* varieties, requires an understanding of the underlying physiological responses and molecular mechanisms of this species. Proteomics has emerged as a powerful tool for the study of plant biological processes [15,16]. Therefore, the primary objective of the work reported herein was to characterize the physiological responses and the changes in the leaf proteomes of two *S. splendens* varieties—the heat-resistant Vista variety and the heat-sensitive King variety—when heat stressed.

## Materials and methods

### Plant materials

Seeds for *S. splendens* Vista and King were obtained from the Zhejiang Hong Yue Flower Co. Ltd., Zhejiang province, China, germinated in 200-cell plug trays that contained a peat/perlite/vermiculite medium (2:1:1, w/w/w) in a greenhouse maintained at 75–80% relative humidity and at 24°C during a 13-h light period and at 18°C during an 11-h period. The white light intensity was 200–300 μmol·m^–2^·s^–1^. After one month, seedlings were transplanted into plastic cups (upper diameter, 6 cm; height, 8 cm) that contained a peat/perlite mixture (8:1, w/w) in an ~60%-shaded area under natural conditions in April of Hangzhou (http://www.hzqx.com), China. Plants were watered weekly with tap water. Healthy, uniform 8-week-old plants that had not blossomed were used for the experiments. These plants were subjected to a 26°C (13-h light)/20°C (11-h dark) temperature/light cycle for a 2-d acclimation period in an Intelligent Climate Incubator (EHI400, Lead-tech USA). The light intensity was 400 μmol·m^–2^·s^–1^ (white light). For each experiment, 30 leaves were randomly chosen and removed from five plants.

### Cell membrane stability (CMS)

CMS was assessed as described [17] with some modifications. Approximately 0.25 g of fully matured leaf discs was rinsed twice with distilled water and then placed in a closed tube with 2 mL of distilled water (three replicates each for the two cultivars). The experimental leaf samples (T1) were held at 42°C in a water bath for 2 h, whereas control samples (C1) were kept at 25°C for the same period. Distilled water (18 mL) was then added to each tube, and the samples were incubated at 25°C for 24 h. Next, the samples were brought to room temperature, and the conductivity of each solution (T1 and C1) was measured. The samples in the tubes were autoclaved at 100°C for 15 min, and the conductivities of these solutions (T2 and C2) were measured. CMS (%) was calculated as {[1 − (*T*1/*T*2)]/[1 − (*C*1/*C*2)]} × 100.

### Heat treatment and measurement of physiological indexes

Acclimated 8-week-old plants were exposed to a 40°C (13-h light)/28°C (11-h dark) cycle in the Intelligent Climate Incubator for 4 d, and then for 2 d to a 26°C (13-h light)/20°C (11-h dark) cycle. The light intensity was 400 μmol·m^–2^·s^–1^. The soil was kept moist at all times. Samples of fully matured leaves were taken at zero time and at 2-, 4-, and 6-d intervals for the heat stressed plants to measure Fv/Fm and SPAD values. Leaf samples (0.2 g) were also frozen in liquid nitrogen and stored at –80°C for CAT, POD, and SOD activity assays and to measure malondialdehyde and proline levels.

Fv/Fm values were measured with a pulse-modulated fluorometer (Min-PAM-2000, Walz, Germany) at 25°C. Before each measurement, the leaf sample was dark-adapted for 20 min. Soil plant analysis development (SPAD) values were determined using a chlorophyll meter (SPAD-502, Minolta, Japan). CAT, POD, and SOD activities and malondialdehyde and proline levels were determined as described [15].

### Protein extraction

Protein was extracted from leaves and quantified as described [18] with minor modifications. A portion (3 g) of each leaf was pulverized with a pestle in a mortar that contained liquid nitrogen and then homogenized in 8 mL of 10% (w/v) trichloroacetic acid, 0.07% (v/v) 2-sulfanylethanol in acetone. Total protein was precipitated for 1 h or overnight at –20°C. The extracts were each centrifuged at 12000 × *g* for 25 min at 4°C. The pellets were washed three times with 0.07% (v/v) 2-sulfanylethanol in acetone, and vacuum dried for 20 min. Each dried powder (20 mg) was suspended in 300 μL of 7 M urea, 2 M thiourea, 4% (w/v) 3-[(3-cholamidopropyl) dimethylammonio]-1-propanesulfonate (CHAPS), 0.75% (w/v) dithiothreitol (DTT), 0.5% (w/v) Biolyte (pH 3.0–10.0, Bio-Rad), 1 mM phenylmethanesulfonyl fluoride, and then shaken vigorously for 1.5 h at room temperature. Insoluble material was removed by centrifugation at 12000 × *g* for 20 min at 20°C. At least three replicates were prepared. Protein concentrations were determined using Bio-Rad Protein Assay kit reagents with bovine serum albumin as the calibration standard [19].

### 2-DE

Each sample contained 350 μg protein in 400 μL of 8 M urea, 2 M thiourea, 2% (w/v) CHAPS, 0.5% (w/v) Biolyte (pH 3–10), 0.75% (w/v) DTT, 0.002% (w/v) bromophenol blue. Each sample was each loaded onto a 17-cm immobilized pH (3–10) gradient strip (Bio-Rad). The strips were hydrated for 12 h at 50 V. Isoelectric focusing used a linear ramp from 0 to 250 V in 15 min, a linear ramp from 250 to 10000 V in 1 h, and 10000 V for 5 h, all at 20°C. After isoelectric focusing, the strips were equilibrated in 50 mM Tris-HCl, pH 8.8, 6 M urea, 20% (v/v) glycerol, 2% (w/v) sodium dodecyl sulfate (SDS), 2% (w/v) DTT, and then in a solution of the same composition that contained 2.5% (w/v) iodoacetamide and no DTT (incubation times were each 15 min). The strips were then each placed onto a 1-mm-thick SDS-PAGE gel (12.5% w/v polyacrylamide) and sealed with 1% (w/v) agarose. Electrophoresis was carried out in a Bio-Rad PROTEAN apparatus at 24 mA/gel. The gels were stained using a modified silver-staining method that is compatible with MS [20]. Image analysis was subsequently performed. These procedures were replicated three times.

### Image acquisition and analysis

The 2-DE gels were scanned using a calibrated densitometer (GS-800, Bio-Rad), and the spot patterns were characterized using PDQuest software (ver. 8.0.1, Bio-Rad). Imaging steps were image filtration, spot detection and measurement, background subtraction, and spot matching. Initially, spots were automatically matched, and the positions of unmatched spots were then manually determined. The molecular mass of each protein was estimated by comparison with those of a standard marker set, and the isoelectric points (pIs) were determined by the spot positions along the immobilized pH gradient strips.

### In-gel protein digestion and MS

Silver-stained protein spots were manually excised from gels, and each was placed into a well of a 96-well microplate. The gel pieces were destained in a solution prepared from a 1:1 (v/v) mixture of 30 mM potassium ferricyanide and 100 mM sodium thiosulfate at room temperature for 20 min, vortexed until destained, washed three times with 200 μL of Milli-Q water (each time for 5 min), and dehydrated in 100 μL of acetonitrile. Then the gel samples were swollen in 50 mM NH_4_HCO_3_, 12.5 ng/μL trypsin (Sigma, Cat. No. 089K6048) at 4°C for 25 min, and at 37°C for more than 12 h. For each digest, the peptides were extracted from the gels twice with 5% trifluoroacetic/50% acetonitrile at room temperature, then suspended in 0.7 μL of 0.2 M α-cyano-4-hydroxy-cinnamic acid (Sigma) in 0.1% trifluoroacetic/50% (v/v) acetonitrile, and dried under a stream of nitrogen. The extracted peptides were subjected to tandem MS using a 4800 MALDI-TOF/TOF Proteomics Analyzer (Applied Biosystems, Foster City, USA). Parent mass peaks with masses between 700 and 3200 Da and a minimum signal-to-noise ratio of 20 were chosen for tandem MS. Combined mass and tandem mass spectra were analyzed using MASCOT (Version 2.1, Matrix Science, London, U.K.) by Data Explorer software (Version 4.5, Applied Biosystems) and searched using the NCBI non-redundant database (version released 20110709, including 14,652,852 sequences and 5,012,444,178 residues) for proteins from *Viridiplantae* (green plants, 88,962 sequences). The search parameters were: (1) peptide molecular mass, 800–4000 Da, mass tolerance for the fragment ion, 0.25 Da; (2) a minimum of one matching peptides; (3) one missed cleavage allowed; and (4) carbamidomethylation of cysteine required, acetylation of the N-terminus allowed, and methionine oxidation allowed. To evaluate protein identification, we considered the percentage of sequence coverage, the observed distribution of matched peptides (an authentic protein hit is often characterized by identification of peptides that are adjacent to one another in the sequence and/or that overlap), the error distribution (around zero), the differences between probability and score distribution for the first and all other candidates, and matches with >90% sequence identity and a maximum e-value of 10^−10^. Using PDQuest software, fold increase and decrease for the proteins from the heat-stressed leaves vs. the control proteins were calculated as treated/control and control/treated for up- and downregulated proteins, respectively. Only the protein spots whose expression intensity treated/control values that exhibited significant changes (>1.5-fold, *p*≤0.05) were included for further analysis.

### GO annotation and protein classification

The UniProt database (http://www.uniprot.org) was searched to determine the functions of the identified proteins. Three independent ontological sets in the *Viridiplantae* taxonomic database were used to annotate and group the proteins according to biological process, molecular function, and cellular compartmentalization.

### Statistical analyses

Each reported datum (CMS and Fv/Fm values, SPAD, CAT, POD and SOD activities, malondialdehyde and proline levels) is the mean ± standard deviations (SD) of three replicates. Statistical analyses were performed by analysis of variance (ANOVA) using the Microsoft Excel Program and comparisons between the mean values were made by least significant difference (LSD) at a 0.05 probability level.

### Quantification of gene expression by quantitative real-time PCR

Specific primers were designed according to the corresponding gene sequences of MS-identified proteins using Primer 3 (Version 0.4.0). All primer information is listed in Additional file 1. Total RNA was extracted from the leaves of Vista and King using Trizol according to the supplier’s recommendation (Invitrogen, Germany). Residual DNA was removed with RNase-free DNase (Fermentas, Canada). Next, 1 μg of total RNA was reverse transcribed using 0.5 μg of Oligo (dT)20, and 200 units of ReverTra Ace (TOYOBO, Japan) following the supplier’s recommendations. Quantitative real-time PCR was performed using the Opticon 2 Real-time PCR Detection System (Bio-Rad, USA). PCR was performed using SYBR Green Supermix (Bio-Rad, USA). The PCR conditions consisted of 40 cycles of denaturation at 95°C for 30 s, annealing at 58°C for 45 s, and extension at 72°C for 30 s. A dissociation curve was generated at the end of each PCR cycle to verify that a single product had been amplified using the software provided with the Opticon 2 Real-time PCR Detection System. To minimize sample variation, mRNA expression of each target gene was normalized to the expression of the housekeeping gene 5.8S ribosomal RNA. All experiments were repeated three times. Quantification of mRNA used the comparative threshold cycle (Ct) method [21]. The Ct value for 5.8S ribosomal RNA (the internal standard) was subtracted from that of the gene of interest to obtain a ΔCt value. The Ct value of the control plant sample was subtracted from the ΔCt value to obtain a ΔΔCt value. Each fold change in expression level relative to that of the control was expressed as 2^–ΔΔCt^.

## Results

### Morphological and physiological changes induced by heat stress

**Figure 1:**
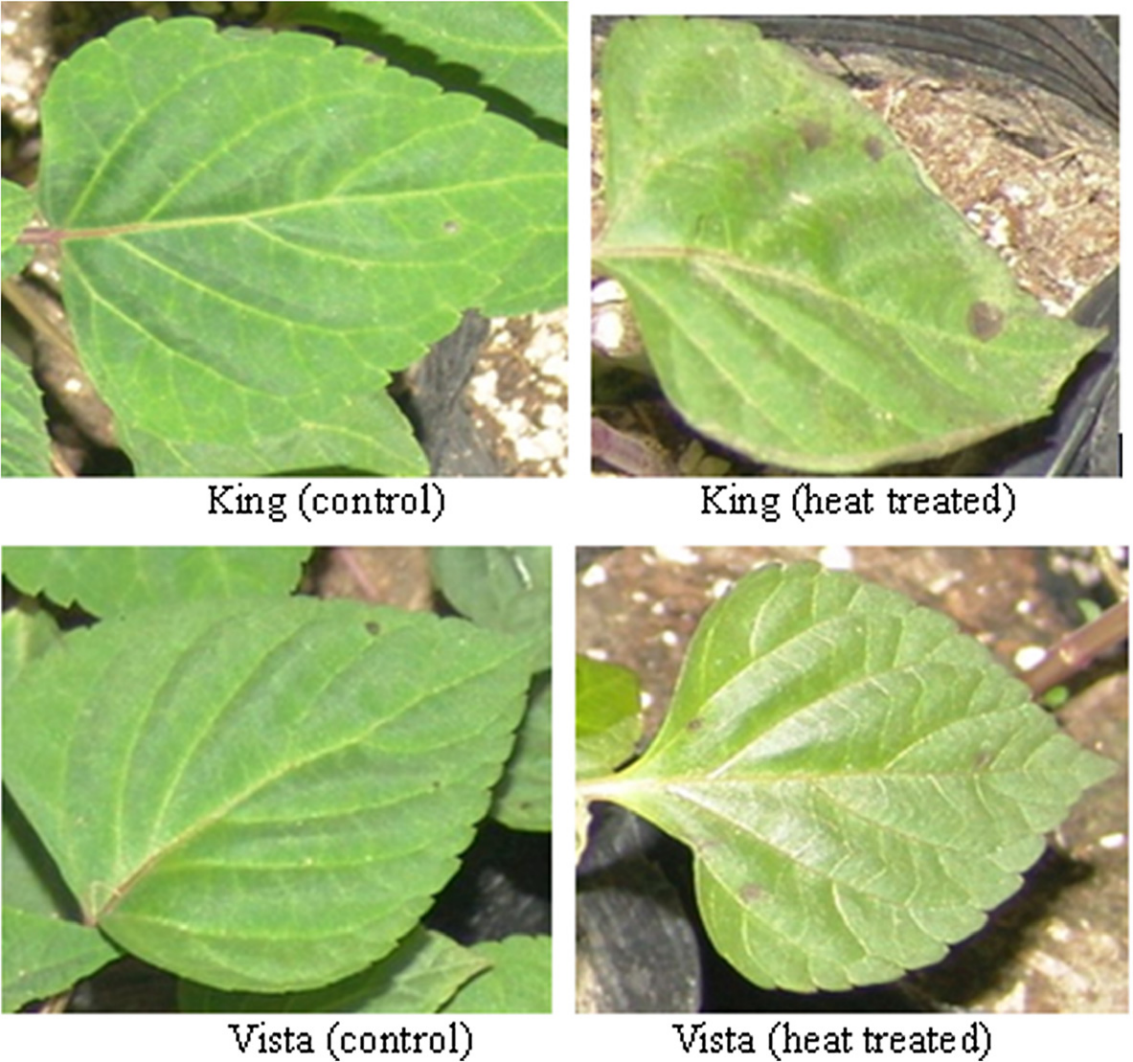
**Heat stress causes marginal burns on leaves of *S. splendens *King.** Acclimated 8-week-old, healthy plants were exposed to a 40°C (13-h light period)/28°C (11-h dark period) cycle in an Intelligent Climate Incubator for 3 d. Control plants were cultivated under a 26°C (light)/20°C (dark) cycle. Light intensity was 400 μmol·m^–2^·s^–1^. The media were kept moist throughout the experiment.

Burn marks, which manifested as reddish-brown spots, were seen on only King leaves induced by heat stress, and some of those leaves also had a scorched-like appearance. Conversely, Vista leaves had only weak filamentous lesions (Figure 1).

**Figure 2:**
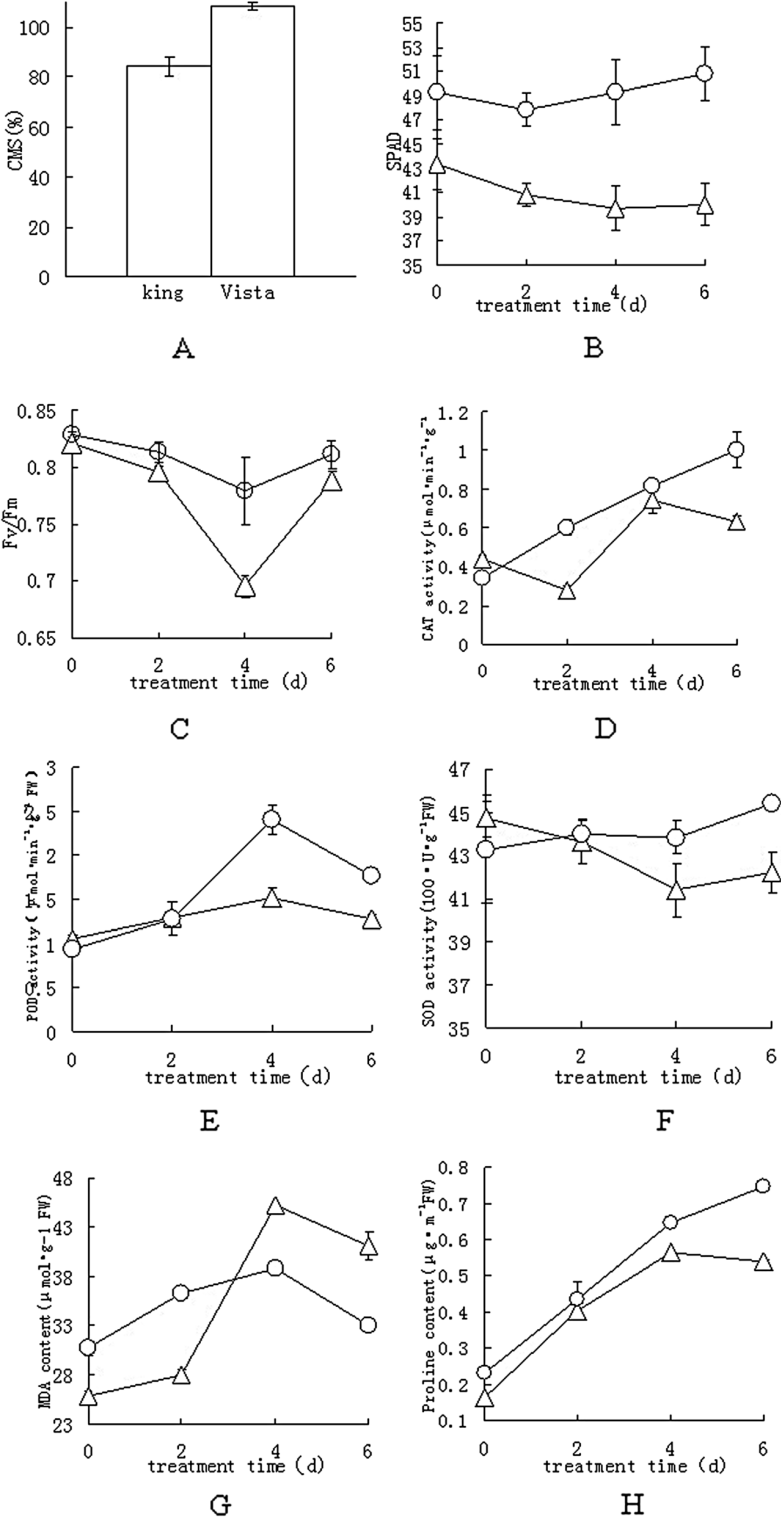
**Biochemical responses of *S. splendens *to heat stress.** Acclimated 8-week-old plants were exposed to a 40°C (13-h light)/28°C (11-h dark) cycle in the Intelligent Climate Incubator for 4 d, and then for 2 d to a 26°C (13-h light)/20°C (11-h dark) cycle. **A**, CMS values. Six independent measurements were made for each test, and each reported value is the mean ± S.E. **B**, SPAD values. **C**, Fv/Fm values. Eight independent measurements were made for each test, and each reported value is the mean ± S.E. **D–H**, Four independent measurements were made for each test, and each reported value is the mean ± S.E. **D**, CAT activity. **E**, POD activity. **F**, SOD activity. **G**, Malondialdehyde content. **H**, Proline content. Symbols: ∆, King; ○, Vista.

Cell membrane stability (CMS) is a standard method by which plant heat tolerance is evaluated [9,22]. After heat stress, differences in the CMS values were recorded for the two *S. splendens* cultivars, with Vista exhibiting a significantly greater CMS value (*p*≤0.05) than King (Figure 2A), which suggests that Vista leaves are more heat tolerant than are King leaves.

Soil plant analysis development (SPAD) values provide relative measures of the chlorophyll content in leaves. Under heat stress, the SPAD value for the King leaves declined with time during the 4 d of stress and did not increase during the recovery period (Figure 2B). Conversely, the SPAD values for the Vista leaves appeared to be unaffected by heat.

The ratio of the variable chlorophyll fluorescence to the maximum fluorescence (Fv/Fm) is a physiological parameter that correlates with heat tolerance [23]. The Fv/Fm values for the leaves of the two species changed when exposed to a temperature of 40°C (Figure 2C). The Fv/Fm values for both species declined during the stress period, but the rate of decrease was more rapid for King leaves than for Vista leaves. However, the Fv/Fm values returned to normal by the end of the recovery period for both cultivars, suggesting that heat stress induced reversible inactivation of PSII reaction centers [24,25].

The activities of the antioxidant enzymes, CAT, POD, and SOD, were affected by heat stress (Figure 2D, E, and F). The Vista CAT and POD activities increased significantly with time, although POD activity decreased during the recovery period. However, the Vista SOD activity never changed significantly (Figure 2F). The King CAT and POD activities increased significantly with time and decreased during the recovery period. Unlike Vista SOD activity, a significant decrease (*p*≤0.05) in SOD activity was found for King leaves subjected to heat stress. The activities of the three enzymes were greater in Vista leaves, especially during the heat-stress period, which indicates that Vista leaves have a greater ability than do King leaves to develop light-protective and oxygen-scavenging mechanisms.

Malondialdehyde (MDA) is a highly reactive three carbon dialdehyde produced as a byproduct of polyunsaturated fatty acid peroxidation and arachidonic acid metabolism. Therefore, measurement of MDA is widely used as an indicator of lipid peroxidation [26]. In addition, Proline accumulation is a common physiological response in many plants in response to a wide range of biotic and abiotic stresses, and it is also widely used as an indicator of stress tolerance in plants [27]. The MDA content significantly increased (*p*≤0.05) in the leaves of both genotypes when heat stressed (Figure 2G) with a greater accumulation in King leaves. The MDA levels decreased in both varieties during the recovery period. Heat treatment also significantly increased (*p*≤0.05) the proline content in the leaves of both genotypes (Figure 2H), although Vista leaves had more proline than did King leaves even during the recovery period.

### Identification of heat stress-responsive proteins by two-dimensional gel electrophoresis (2-DE) and tandem mass spectrometry (MS)

**Figure 3:**
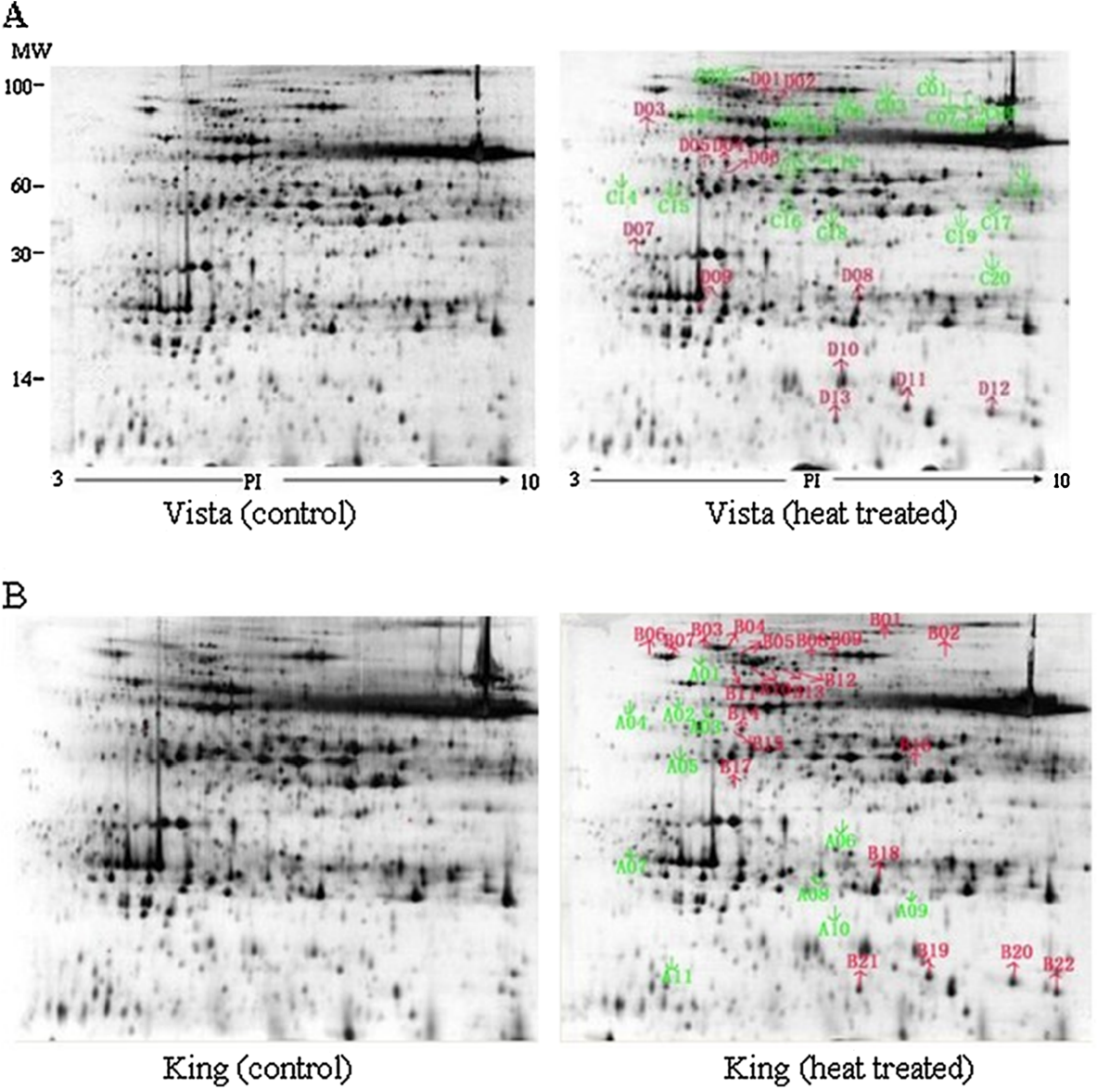
**2-DE gel analysis of the Vista and King leaf proteomes.** Proteins (350 μg) extracted from *S. splendens* leaves were separated in the first dimension by isoelectric focusing (pH 3–10) and in the second dimension by SDS-PAGE (12.5% (w/v) acrylamide). Proteins were visualized by silver staining. The 33 spots that showed significant volume changes caused by heat stress are labeled for both cultivars. **A**, Left gel, control Vista proteome. Right gel, heat-treated Vista proteome. **B**, Left gel, control King proteome. Right gel, heat-treated King proteome.

**Figure 4:**
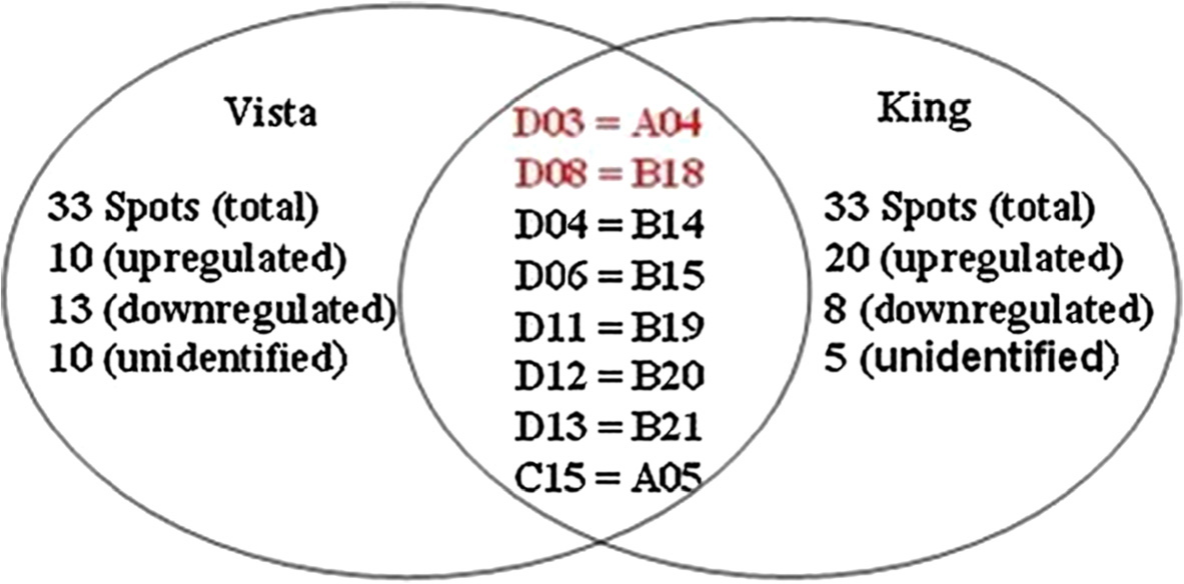
**Venn diagram of the differentially regulated Vista and King proteins.** The numbers of differentially expressed proteins found in the cultivar proteomes are given. For differentially regulated proteins found in both proteomes, the spot numbers for Vista proteins are given on the right and the spot numbers for the corresponding King proteins are given on the left. The proteins for the spot numbers in red were not identified.

We characterized the changes in the protein profiles of the leaves when heat stressed. Leaves from control and heat-stressed plants were harvested, and total protein was resolved and separated by 2-DE. Representative 2-DE gels are shown in Figure 3. The pI values of the spots ranged from 3.2 to 8.7, and the molecular masses ranged from 7.8 to 102.2 kDa. Comparing 2-DE gels from the control and 40°C-treated samples of two *S. Splendens* cultivars, showed many differences in protein abundance. The intensities of 33 spots were significantly altered by heat stress, for both cultivars (Figures 3 and 4) and were subsequently subjected to tandem MS (Table 1 and Additional file 2). Of these proteins, 23 Vista and 28 King proteins could be identified, whereas 10 Vista and 5 King proteins could not be identified. Among the identified proteins, 10 Vista and 20 King proteins were upregulated, whereas 13 and 8 were downregulated, respectively. Eight of the differentially regulated proteins (spots D03, D08, B14, D06, D11, D12, D13, C15 for Vista and spots A04, B18, D04, B15, B19, B20, B21, A05 for King) were found in the proteomes of both cultivars (Figure 4), although spots D03 (=A04) and D08 (=B18) could not be identified.

**Table 1:** Differentially expressed proteins and their relative changes induced by heat stress in Vista leaves as determined by 2-DE

**Spot no.**^***a***^	**Protein**	**NCBI accession no.**^***b***^	**Reference organism**	**kDa/pI**^***c***^	**Score**^***d***^	**MASCOT peptide matches**	**SC (%)**^***e***^	**Average fold change (Mean ± SD)**^***f***^	**Biological process**^***g***^	**Cellular compartment**^***h***^	**Molecular function**^***i***^
Upregulated											
D01	Mthsc70-1	gi|297798080	*Arabidopsis lyrata subsp. lyrata*	73.37/5.52	169	14	20	2.42 ± 0.22	Protein folding	Unknown	ATP binding
D02	Mthsc70-1	gi|297798080	*Arabidopsis lyrata subsp. lyrata*	73.37/5.52	214	11	16	2.11 ± 0.13	Protein folding	Unknown	ATP binding
D04	Chloroplast ribulose-1,5-bisphosphate carboxylase activase	gi|225580059	*Solenostemon scutellarioides*	47.71/8.16	219	11	23	2.81 ± 0.34	Stress response	Chloroplast	ATP binding
D05	Chloroplast ribulose-1,5-bisphosphate carboxylase activase	gi|225580059	*Solenostemon scutellarioides*	47.71/8.16	109	9	21	2.60 ± 0.16	Stress response	Chloroplast	ATP binding
D06	Chloroplast ribulose-1,5-bisphosphate carboxylase activase	gi|225580059	*Solenostemon scutellarioides*	47.71/8.16	114	3	9	3.60 ± 0.47	Stress response	Chloroplast	ATP binding
D09	Chlorophyll a/b binding protein	gi|398599	*Amaranthus hypochondriacus*	28.71/5.68	98	3	17	1.89 ± 0.17	Photosynthesis	Chloroplast	Metal ion binding
D10	Cytochrome B6-F complex iron sulfur subunit 2	gi|146454656	*Sonneratia caseolaris*	16.83/5.75	81	3	32	1.97 ± 0.09	Electron transport	Chloroplast	Oxidoreductase
D11	Class-1 LMW heat shock protein	gi|25044839	*Ananas comosus*	17.56/6.77	182	3	16	7.47 ± 0.43	Stress response	Nucleus	Unknown
D12	Class-1 LMW heat shock protein	gi|25044839	*Ananas comosus*	17.56/6.77	211	4	21	9.16 ± 0.33	Stress response	Nucleus	Unknown
D13	Unknown	gi|224286712	*Picea sitchensis*	13.08/6.34	80	1	13	2.24 ± 0.12	Unknown	Unknown	Unknown
Downregulated											
C02	Predicted protein	gi|224069527	*Populus trichocarpa*	90.78/5.05	131	9	16	−1.83 ± 0.11	Unknown	Unknown	Unknown
C03	Ribulose-1,5-bisphosphate carboxylase subunit	gi|46326312	*Salvia coccinea*	50.28/6.23	247	15	29	−1.74 ± 0.07	Photosynthesis	Chloroplast	Transferase
C04	Atsco1 /Cpef-g	gi|297837151	*Arabidopsis lyrata subsp. lyrata*	86.61/5.52	178	13	19	−1.53 ± 0.08	Unknown	Intracellular	GTP binding
C05	hypothetical protein	gi|225426927	*Vitis vinifera*	88.91/5.09	157	15	23	−1.79 ± 0.14	Unknown	Unknown	Unknown
C10	FtsH-like protein Pftf precursor	gi|4325041	*Nicotiana tabacum*	74.51/6.00	271	14	27	−1.70 ± 0.24	Protein catabolic process	Membrane	ATP binding
C12	Glucose-1-phosphate adenylyltransferase	gi|297835714	*Arabidopsis lyrata subsp. lyrata*	57.65/6.54	81	7	13	−1.57 ± 0.25	Starch biosynthesis	Unknown	ATP binding
C13	GDP-D-mannose-3′,5′-epimerase	gi|223469963	*Ribes nigrum*	42.94/5.88	207	10	34	−1.68 ± 0.09	Cellular metabolic process	Unknown	Catalytic activity
C15	Unknown	gi|217071972	*Medicago truncatula*	19.00/7.74	97	6	44	−1.94 ± 0.33	Unknown	Unknown	Unknown
C16	Putative actin protein	gi|300429861	*Salvia miltiorrhiza*	41.89/5.38	182	11	43	−1.74 ± 0.14	Transcription regulation	Nucleus	Protein binding
C17	NAD dependent epimerase/dehydratase, putative	gi|255542956	*Ricinus communis*	42.72/8.52	179	6	18	−2.06 ± 0.26	Cellular metabolic process	Unknown	Catalytic activity
C18	Degp1	gi|338858724	*Arabidopsis halleri subsp. gemmifera*	21.93/5.75	73	3	24	−1.53 ± 0.06	Stress response	Chloroplast	Serine-type endopeptidase activity
C19	Unnamed protein product	gi|296086699	*Vitis vinifera*	31.31/5.74	135	5	16	−1.53 ± 0.24	Unknown	Unknown	Unknown
C20	Ferredoxin--NADP reductase, putative	gi|255538962	*Ricinus communis*	40.74/8.7	113	7	17	−1.71 ± 0.16	Electron transport	Membrane	Protein binding

^*a*^As indicated in Figure 2A. ^*b*^NCBI accession number. ^*c*^Theoretical isoelectric points (pI values) and molecular masses (kDa). ^*d*^Mascot score. ^*e*^Sequence coverage. ^*f*^Spot abundance is expressed as the ratio of the intensities of the heat-stressed to the corresponding control proteins. Positive values, upregulated; negative values, downregulated. ^*g–i*^Biological processes, cellular compartments, and molecular functions were inferred from those reported in the UniProt database.

### Functional classification and subcellular locations of the identified proteins

Identification of the proteins that are differentially expressed is an important step towards understanding the mechanisms underlying stress responses and adaptation in plants. To further characterize the differentially expressed proteins, the identified proteins were grouped according to their biological processes, cellular locations, and functions using the Gene Ontology (GO) annotation in the Viridiplantae taxonomic databases (Tables 1 and 2). The identified proteins are involved in photosynthesis, metabolism, protein folding, and stress responses; however, functions could not be assigned to most of the differentially expressed proteins.

**Table 2:** Differentially expressed proteins and their relative changes induced by heat stress in King leaves as determined by 2-DE

**Spot no.**^***a***^	**Protein**	**NCBI accession no.**^***b***^	**Reference organism**	**kDa/pI**^***c***^	**Score**^***d***^	**MASCOT peptide matches**	**SC (%)**^***e***^	**Average fold change (Mean ± SD)**^***f***^	**Biological process**^***g***^	**Cellular compartment**^***h***^	**Molecular function**^***i***^
Upregulated											
B01	Hypothetical protein VITISV_009951	gi|147842424	*Vitis vinifera*	89.30/5.28	190	17	24	1.89 ± 0.19	Unknown	Unknown	Unknown
B03	Heat shock protein 83	gi|1708314	*Ipomoea nil*	81.06/4.95	296	20	27	1.62 ± 0.29	Stress response	Cytoplasm	Unfolded protein binding
B04	Heat shock protein 83	gi|1708314	*Ipomoea nil*	81.06/4.95	300	16	22	1.61 ± 0.14	Stress response	Cytoplasm	Unfolded protein binding
B05	ER-binding protein	gi|57639078	*Malus pumila*	73.76/5.14	113	6	11	2.51 ± 0.37	Unknown	Unknown	ATP binding
B06	Unknown	gi|148906951	*Picea sitchensis*	75.93/5.58	147	10	19	1.52 ± 0.25	Unknown	Unknown	Unknown
B07	Unknown	gi|148907083	*Picea sitchensis*	75.84/5.46	168	9	17	1.54 ± 0.16	Unknown	Unknown	Unknown
B08	Transketolase, putative	gi|255541252	*Ricinus communis*	81.62/6.52	88	8	12	2.61 ± 0.09	Energy reserve metabolic process	Cytosol	Unknown
B09	Precursor of protein cell division protease ftsh-like protein	gi|224065699	*Populus trichocarpa*	75.74/6.0	238	19	29	6.31 ± 0.47	Cell division	Membrane	ATP binding
B10	Predicted protein	gi|224098390	*Populus trichocarpa*	71.62/5.14	305	18	25	3.30 ± 0.35	Unknown	Unknown	Unknown
B11	Predicted protein	gi|224098390	*Populus trichocarpa*	71.62/5.14	157	16	23	9.55 ± 1.07	Unknown	Unknown	Unknown
B12	Predicted protein	gi|326499406	*Hordeum vulgare subsp. vulgare*	71.62/5.09	219	14	18	2.12 ± 0.43	Unknown	Unknown	Unknown
B13	Cytosolic heat shock 70 protein	gi|2642648	*Spinacia oleracea*	71.84/5.09	82	12	22	9.23 ± 0.97	Stress response	Cytoplasm	ATP binding
B14	Chloroplast ribulose-1,5-bisphosphate carboxylase/oxygenase activase	gi|225580059	*Solenostemon scutellarioides*	47.71/8.16	219	11	23	1.94 ± 0.12	Stress response	Chloroplast	ATP binding
B15	Chloroplast ribulose-1,5-bisphosphate carboxylase/oxygenase activase	gi|225580059	*Solenostemon scutellarioides*	47.71/8.16	114	3	9	1.57 ± 0.28	Stress response	Chloroplast	ATP binding
B16	Predicted protein	gi|224056853	*Populus trichocarpa*	38.65/7.56	148	3	13	2.26 ± 0.14	Unknown	Unknown	Unknown
B17	Unknown	gi|118486395	*Populus trichocarpa*	18.46/5.08	85	3	18	9.61 ± 0.55	Unknown	Unknown	Unknown
B19	Class-1 low-molecular-weight heat shock protein	gi|25044839	*Ananas comosus*	17.56/6.77	182	3	16	7.91 ± 0.42	Stress response	Unknown	Unknown
B20	Class-1 low-molecular-weight heat shock protein	gi|25044839	*Ananas comosus*	17.56/6.77	211	4	21	6.86 ± 0.49	Stress response	Unknown	Unknown
B21	Unknown	gi|224286712	*Picea sitchensis*	13.08/6.34	80	1	13	1.95 ± 0.13	Unknown	Unknown	Unknown
B22	Unknown	gi|224286712	*Picea sitchensis*	13.08/6.34	95	1	13	1.99 ± 0.23	Unknown	Unknown	Unknown
Downregulated											
A01	RuBisCO large subunit-binding protein subunit alpha, chloroplastic	gi|1710807	*Pisum sativum*	62.00/5.15	103	10	19	−1.87 ± 0.17	Photosynthesis	Chloroplast stroma	ATP binding
A02	ATP synthase beta subunit	gi|123325679	*Luvunga sp. TT 364*	50.00/5.1	77	8	26	−1.89 ± 0.26	ADP biosynthetic process	Mitochondrial	ATP binding
A03	ATP synthase CF1 beta subunit	gi|306485983	*Franklinia alatamaha*	51.00/5.35	417	19	59	−1.66 ± 0.11	ADP biosynthetic process	Chloroplast	ATP binding
A05	Unknown	gi|217071972	*Medicago truncatula*	19.00/7.74	97	6	44	−2.28 ± 0.28	Unknown	Unknown	Unknown
A06	Similar to Probable pyridoxal biosynthesis protein PDX1	gi|225434584	*Vitis vinifera*	33.33/5.94	263	12	28	−1.71 ± 0.08	Pyridoxal phosphate biosynthetic process	Unknown	Catalytic activity
A09	Hypothetical protein LOC100382672	gi|293333399	*Zea mays*	32.02/9.24	91	4	13	−1.55 ± 0.33	Unknown	Unknown	Unknown
A10	Hypothetical protein	gi|225437581	*Vitis vinifera*	31.73/7.71	105	4	19	−2.22 ± 0.21	Unknown	Unknown	Unknown
A11	Hypothetical protein	gi|110742537	*Arabidopsis thaliana*	23.17/4.63	74	2	10	−1.85 ± 0.12	Unknown	Unknown	Unknown

^*a*^As indicated in Figure 2A. ^*b*^NCBI accession number. ^*c*^Theoretical isoelectric points (pI values) and molecular masses (kDa). ^*d*^Mascot score. ^*e*^Sequence coverage. ^*f*^Spot abundance is expressed as the ratio of the intensities of the heat-stressed to the corresponding control proteins. Positive values, upregulated; negative values, downregulated. ^*g–i*^Biological processes, cellular compartments, and molecular functions were inferred from those reported in the UniProt database.

#### Proteins involved in protein folding and stress response

Misfolded proteins often accumulate in plant cells that are stressed. Plants may employ two strategies to counteract this phenomenon. The proteins are refolded or degraded [28]. Hsp70 helps maintain the structural/functional integrity of proteins and facilitates intercellular transport of enzymes during osmotic stress [29,30]. We identified two upregulated Vista proteins (spots D01 and D02) as isoforms of the mitochondrial Hsp70-1. This Hsp binds zinc and ATP and responds to cadmium-ion and abiotic stresses. It is located in the mitochondrial matrix, cell walls, and plasma membranes.

Abiotic stresses usually cause protein dysfunction [9,22]. For Vista, six of the identified proteins (spots D04, D05, D06, D11, D12 and C18) are involved in stress response. All of these proteins, with the exception of spot C18 were upregulated. Spots D04, D05, and D06 are ribulose-1,5-bisphosphate carboxylase oxygenase Rubisco (RuBisCO) activases which are required to allow the rapid formation of the critical carbamate in the active site of RuBisCO. Spots D11 and D12 are class-1 low-molecular-weight Hsps (sHsps), which are ubiquitously found in prokaryotic and eukaryotic cells in response to heat and other stresses. Spot C18 is DEGP1 (Periplasmic serine endoprotease) protease, a chloroplast-targeted protease that, because it degrades the lumenal proteins plastocyanin and OE33 (a oxygen-evolving complex subunit), may act as a general-purpose protease in the thylakoid lumen. It is also involved in the degradation of the D1 protein of PSII, thereby helping to repair photoinhibition-induced damage to PSII [31,32].

Seven of the identified King proteins (spots B03, B04, B13, B14, B15, B19 and B20) are involved in stress response. All were upregulated. Spots B03 and B04 are Hsp90-type Hsps for which signal transduction proteins such as steroid hormone receptors and signaling kinases are substrates. Hsp90 manages protein folding but also is involved in signal-transduction networks, cell-cycle control, protein degradation, and protein trafficking [9,33]. Spot B13 is a Hsp70-type protein, which help prevent protein aggregation, assist in the refolding of non-native proteins under both normal and stress conditions, are involved in protein import and translocation processes, and facilitate proteolytic degradation of unstable proteins by targeting such proteins to lysosomes or proteasomes [9,34]. Spots B14, B15, B19, and B20 are the same proteins as the respective spots D04, D06, D11, and D12 from Vista.

#### Proteins involved in photosynthesis

A well-known consequence of heat stress in plants is damage caused by a heat-induced imbalance between photosynthesis and respiration [22]. Two identified Vista proteins (spots D09 and C03) and one King protein (A01) are photosynthesis proteins. Vista spot D09 is the chlorophyll a/b-binding protein, which was upregulated by heat stress. Chlorophyll a/b-binding proteins are the main components of the light-harvesting complex, whose main function is light-capture and efficient energy transfer to photosynthetic reaction centers. Spot C03 is the large subunit of RuBisCO, which contains two types of chains, namely the large and small subunits. In plants, the large subunit is encoded in chloroplast DNA and the small subunit in nuclear DNA [35]. Spot A01 is the large subunit–binding protein, which is implicated in the assembly of RuBisCO in higher plant chloroplasts, and is likely to be encoded in nuclear DNA [36]. Spots C03 and A01 were both downregulated by heat stress.

#### Proteins involved in metabolism

For Vista, identified proteins were those involved in catabolic processes (spot C10), starch biosynthesis (spot C12), and cellular metabolic processes (spots C13 and C17). These proteins were all downregulated by heat stress. One of identified King proteins (spot B08) is a putative transketolase, an enzyme found in the pentose phosphate pathway in animals and the Calvin cycle in photosynthetic organisms and, as such, would be involved in an energy-reserve metabolic process.

### Correlation between mRNA and protein expression

**Figure 5:**
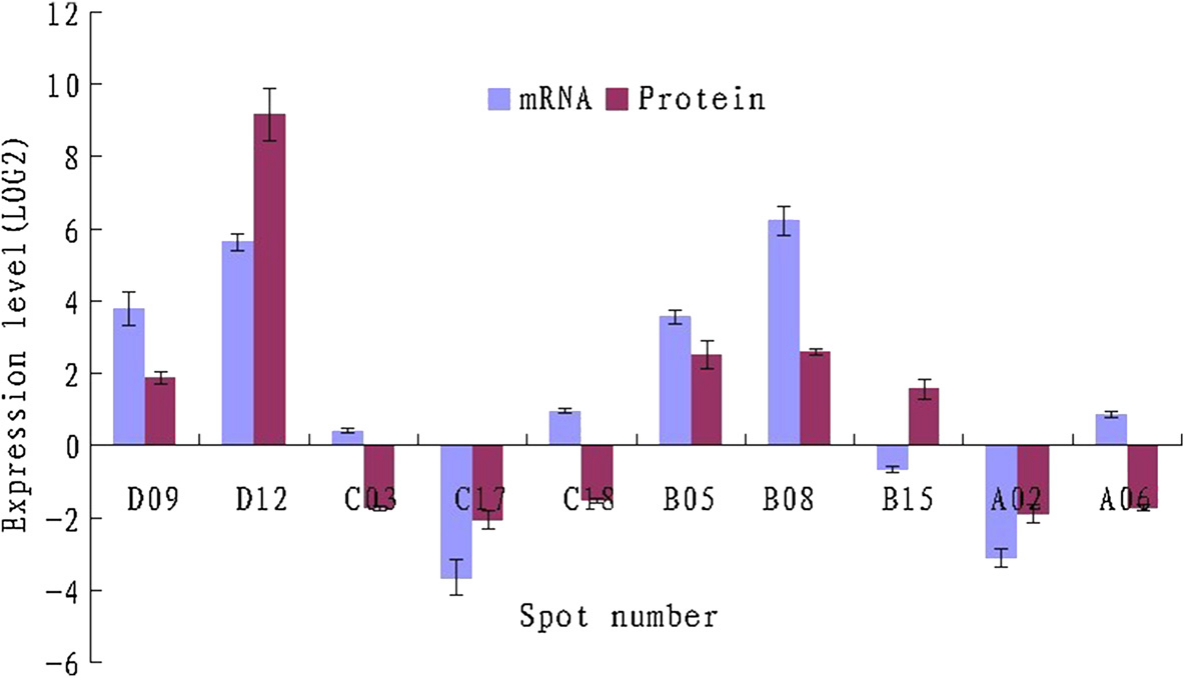
**Comparison of the mRNA and protein expression levels for ten genes.** Quantitative real-time PCR was performed using gene-specific primers (Additional file 1) and SYBR Green Supermix. The protein and mRNA log2 values for the ratios of treated samples to the corresponding controls (CK) are plotted. The relative gene expression was evaluated using the ΔΔCt method. D09, Chlorophyll a/b binding protein; D12, Class-1 LMW heat shock protein; C03, Ribulose-1,5-bisphosphate carboxylase subunit; C17, NAD dependent dehydratase; C18, Degp1; B05, ER-binding protein; B08, Transketolase; B15, Ribulose-1,5-bisphosphate carboxylase activase; A02, ATP synthase beta subunit; A06, Pyridoxal biosynthesis protein PDX1.

To correlate protein levels with corresponding mRNA levels, the transcriptional activities of ten genes from two varieties were assessed. The mRNA transcript of 5.8S ribosomal RNA gene was used as the control. In general, the changes in mRNA abundance didn’t correlate significantly with the changes in expression of their corresponding proteins, excepting those for Chlorophyll a/b binding protein (spot D08), Class-1 LMW heat shock protein (spot D12), NAD dependent dehydratase (spot C17), ER-binding protein (spot B05), Transketolase (spot B08) and ATP synthase (spot A02) (Figure 5). For Vista, mRNA abundance of RuBisCO and Degp1 was elevated, although these two proteins expression was downregulated. Conversely, RuBisCO activase mRNA expression had decreased in King variety, whereas RuBisCO activase expression had increased. It would be possible that mRNA-protein expression correlation varies for different genes. The complicated biological processes, such as transcriptional splicing, post-transcriptional splicing, translational modifications, translational regulation, and protein complex formation, might affect the relative quantities of mRNA and protein of various genes to various degrees. These data, however, provide transcriptional information complementary to the differentially expressed proteins detected by the proteomic study.

## Discussion

Plants use different mechanisms to survive when the temperature is elevated. Such mechanisms include long-term evolutionary phenological and morphological adaptations and short-term avoidance or acclimation mechanisms [12]. When plants are heat stressed, the initial stress responses (e.g., osmotic or ionic effects, or membrane fluidity) trigger additional signaling processes and transcription controls, which activate stress-responsive mechanisms to reestablish homeostasis and protect and repair damaged proteins and membranes. Inadequate responses by one or more of the signaling and gene-activation processes might ultimately result in irreversible perturbation of cellular homeostasis and the destruction of functional and structural proteins and membranes, leading to cell death [37,38]. Some major tolerance responders, including ion transporters, osmoprotectants, free-radical scavengers, abundant stress responsive proteins, and factors involved in signaling cascades and transcriptional control are essential to counteract the effects of heat stress [9].

Here we determined how cultivars Vista and King differed in their response to heat stress. As seen by their morphological and physiological traits (Figures 1 and 2), Vista is indeed more resistant to heat stress, which is also concluded from the capacity of light-protection and oxygen-scavenging (Figure 2). Natarajan and Keuhny [39] reported that morphological traits including short stature, greater total leaf area, an extensive root system, and the physiological traits of stomatal conductance, greater transpiration, are characteristic of *S. splendens* heat-tolerant cultivars. In addition, it was also verified that Vista leaves contained greater concentrations of sucrose and raffinose, which helps stabilize the membrane lipid bilayer and/or act as osmoprotectants. In our study, Vista leaves had more proline than King leaves (Figure 2G).

Moreover, we identified 23 Vista and 28 King proteins (Figure 4) that may function in *S. splendens* heat response (Tables 1 and 2). Most of them are differentially expressed proteins between two cultivars under heat stress. As far as heat-tolerant Vista is concerned, these identified proteins may play more important role in dealing with the adversity of heat stress. For example, the majority of the identified proteins are involved in photosynthesis (spots D09 and C03), metabolism (spot C10, C12, C13 and C17), protein processing (spots D01 and D02), and stress response (D04, D05, D06, D11, D12 and C18), indicating that these processes cooperate to reestablish cellular homeostasis following heat stress for heat tolerant *S. splendens*. Natarajan and Keuhny [39] also reported that expression of a 27-kDa Hsp may be responsible for heat tolerance in Vista leaves. In addition, eight proteins were found in the proteomes of both cultivars, suggesting that Vista and King leaves share certain heat-response mechanisms. For example, Vista spots D11 and D12 are the same as spots B19 and B20 from King, respectively. These two pairs of proteins belong to small heat shock proteins (sHsps), which are respond to many different environmental stresses, including heat, cold, drought, salinity, and oxidation. sHsps abundance in plants and their abilities to bind and stabilize denatured proteins suggest that they are important for plant-acquired tolerance to heat stress [40-42].

**Figure 6:**
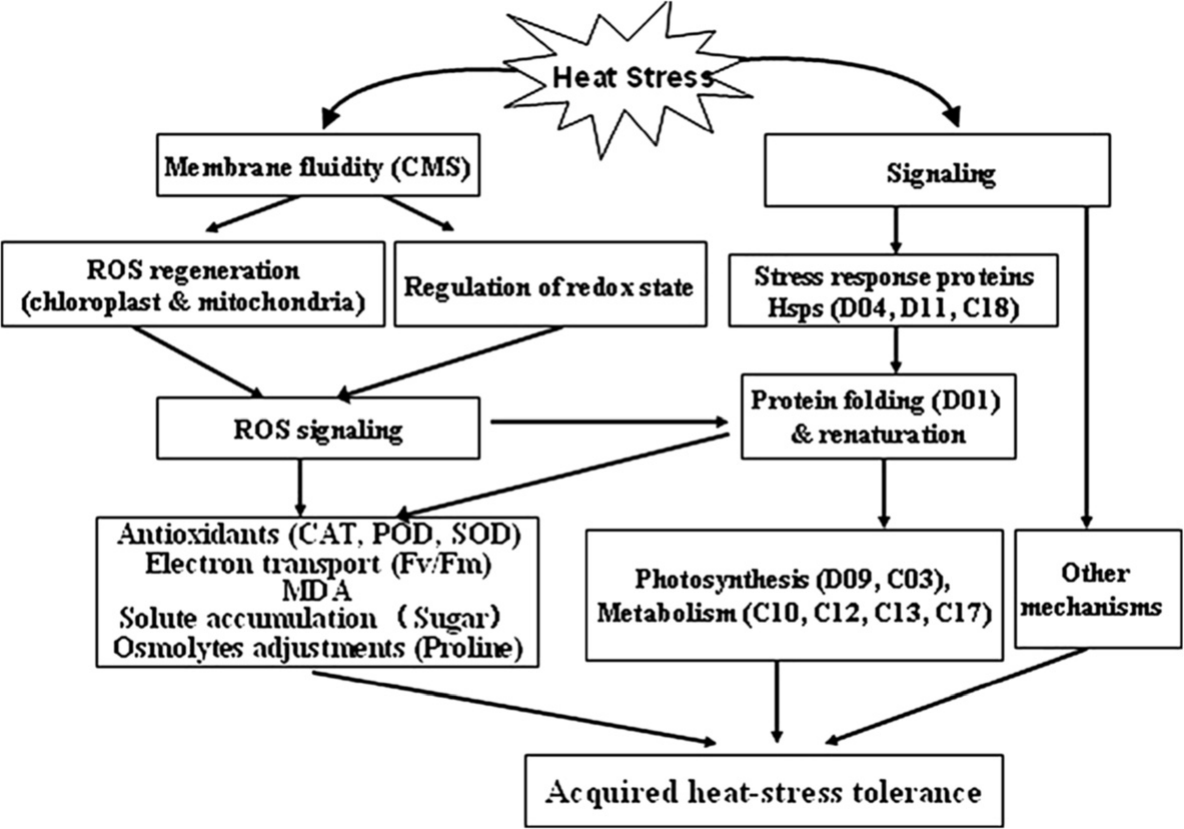
**Possible heat-stress-tolerance system in *S. splendens.*** Initial effects of heat stress are on the plasmalemma and cause electrolyte leakage, ROS production, and oxidative damage. Signaling caused by these effects at the genomic level can lead to increased production of antioxidants and osmolytes that correct for perturbations in the cell-water balance. Production of protective enzymes and antioxidants results in less oxidative damage. The majority of the identified proteins are involved in photosynthesis, metabolism, protein processing, and stress response, indicating that many processes work together to reestablish cellular homeostasis after heat stress.

Thermotolerance refers to the ability of a plant to cope with excessively high temperatures [22]. The physical and metabolic changes enable *S. splendens* to cope with the adversity of heat stress (Figure 6). Initially, membrane-lipid-bilayer fluidity increases, which can cause electrolyte leakage, ROS production, and oxidative damage. ROS may damage cellular components and act as signaling molecules leading to the expression of antioxidant enzymes (Hsps) and a rebalancing of osmolyte concentrations (Proline), that correct for perturbations in the cell-water balance. ROS production in chloroplasts and mitochondria is of great importance for signaling as well, as production of protective enzymes and antioxidants [38], which results in less oxidative damage [43]. Meanwhile, many heat stress-induced proteins, especially Hsps, play a role in stress signal transduction, protecting and repairing damaged proteins and membranes, protecting photosynthesis as well as regulating cellular redox state. However, sequence information for the *S. splendens* genome is limited, and for many of the differentially regulated proteins, the MS/MS data could not be correlated with sequence information available in the Viridiplantae database. This limitation was especially true for the Vista proteins. Probably, many different mechanisms are involved in *S. splendens* heat tolerance. Additional studies are needed to comprehensively delineate the *S. splendens* proteins that are selectively up- or down-regulated in response to heat stress.

## Conclusions

To our knowledge, this is the first proteomic study in leaves from *S. splendens*. In the present study, the responses by two *S. splendens* variants and changes to their proteomes induced by heat were assessed to gain a comprehensive understanding of heat-tolerance mechanisms. A 40°C treatment for 4 d induced reversible inactivation of PSII reaction centers and increased the production of enzymes that protect against oxidative damage. Vista leaves were better able to develop light-protective and anti-ROS mechanisms than were King leaves. More than 1213 protein spots were reproducibly detected on each 2-D gel, with 33 proteins in both variants differentially up- or downregulated by heat stress. Of the 33 proteins, 23 Vista and 28 King proteins were identified, and the two varieties both contained 8 of these proteins. Therefore, Vista and King share some of the heat-tolerant mechanisms. The identified proteins were grouped according to their biological processes, molecular functions, and cellular locations using the GO annotation in the Viridiplantae taxonomic databases. Most are involved in photosynthesis, metabolism, protein processing, and stress response, indicating that many processes work together to establish a new cellular homeostasis when heat stressed. A possible thermotolerance mechanisms model of *Salvia Splendens* is presented in Figure 6. Future work should integrate genomic, proteomic, and metabolomic approaches to achieve a comprehensive knowledge of the sophisticated and fine-tuned thermotolerant molecular mechanisms in response to heat stress.

## Competing interests

The authors declare that they have no competing interests.

## Authors’ contributions

Conceived and designed the experiments: HL GZS XPF QJF. Performed the experiments: HL XPF QJF KKH HY YZ LZ. Analyzed the data: HL XPF YC LJ SLR. Wrote the paper: HL XPF. All authors read and approved the final manuscript.

## Supplementary Material

Additional file 1 Primers used for real-time PCR. Click here for file

Additional file 2 The detailed information about the proteins identified by MS/MS. Click here for file
