# Correction: Heat stress-induced response of the proteomes of leaves from Salvia splendens vista and king

**DOI:** 10.1186/1477-5956-11-35

**Published:** 2013-07-25

**Authors:** Hui Liu, Guozheng Shen, Xianping Fang, Qiaojuan Fu, Kangkang Huang, Yi Chen, Hong Yu, HengMu Zhang, Yun Zhao, Le Zhang, Liang Jin, Songlin Ruan

**Affiliations:** 1 Institute of Horticulture, Hangzhou Academy of Agricultural Sciences, 310024, Hangzhou, China; 2 Institute of Biology, Hangzhou Academy of Agricultural Sciences, 310024, Hangzhou, China; 3 Institute of Crop Science, College of Agriculture & Biotechnology, Zhejiang University, 310029, Hangzhou, China; 4 Experiment Center, Hangzhou Academy of Agricultural Sciences, 310024, Hangzhou, China; 5 Institute of Virology and Biotechnology, Zhejiang Academy of Agricultural Sciences, 310021, Hangzhou, China

## Correction

Upon publication of the article entitled “Heat stress-induced response of the proteomes of leaves from Salvia splendens Vista and King” [1] the authors recognised that Dr Heng-Mu Zhang had been omitted from the author list.

The co-authors would like to apologise for this omission. All authors have agreed to the addition of Dr Hen-Mu Zhang to the revised author list as shown above, for his provision of the original raw experimental materials, contribution to the design of the experimental procedure and quantitative experiments of the plant physiology and biochemistry index.

## References

[B1] Liu H Shen G Fang X Fu Q Huang K Chen Y Yu H Zhao Y Zhang L Jin L Ruan S Heat stress-induced response of the proteomes of leaves from Salvia splendens Vista and King Proteome Science 2013 11 25 10.1186/1477-5956-11-25 23773552 PMC3720558

