# Supplementary material for: Heat stress-induced response of the proteomes of leaves from Salvia splendens Vista and King
Source: Proteome Sci. 2013 Jun 18;11:25. doi: 10.1186/1477-5956-11-25 (PMC3720558; doi:10.1186/1477-5956-11-25)
Supplement: Additional file 2 — The detailed information about the proteins identified by MS/MS. [file 1477-5956-11-25-S2.pdf]

Supplemental Data 2. Primers used for real-time PCR

Gene	Spot no.	Gene symbol	Forward primer	Reverse primer
**Vista variety**				
Chlorophyll a/b binding protein	D09	X74732	TGGGTTTGATTGAGGGCTAC	GGTGATCGGAAAGGTTCTCA
Class-1 LMW heat shock protein	D12	AY098528	CCGCATAGATTGGAAGGAGA	GAACCTGCGTAGGAACTTGC
Ribulose-1,5-bisphosphate carboxylase subunit	C03	AY570407	CACAGGCTGAAACAGGTGAA	CGGCAATAATGAGCCAAACT
NAD dependent dehydratase	C17	XM_002512495	GCAGGCAAAAGGAGCACTAC	GGTCTGATTCACCTGGCAAT
Degp1	C18	AB642507	GAGGTACCACAAGGCTCTGG	AAGATCCGCAGAGACTCCAA
**King variety**				
ER-binding protein	B05	AY729667	AGCGACAGATCGACGAGATT	GAGCGACATCAAGGAGAAGG
Transketolase	B08	XM_002511644	CCTTTCCATGTTCCAGAGGA	GTAGCATCTGCTGGGCTTTC
Ribulose bisphosphate carboxylase activase	B15	FJ787730	CAACAACCAGATGGTCAACG	TACCCTTGCACACACCGATA
ATP synthase beta subunit	A02	EF118880	TATCCGTATTTGGCGGAGTC	CCATAGTTAGGGCCGTCAAA
pyridoxal biosynthesis protein PDX1	A06	XM_002278355	TGATGATGCAGTTGGGTTGT	CTGACCGATTCGCATACCTT
**Reference gene**				
5.8S ribosomal RNA		AF477788	CAACGGATATCTCGGCTCTC	TTGCGTTCAAAGACTCGATG
